# Why True Believers Make the Ultimate Sacrifice: Sacred Values, Moral Convictions, or Identity Fusion?

**DOI:** 10.3389/fpsyg.2021.779120

**Published:** 2021-11-15

**Authors:** Francois Alexi Martel, Michael Buhrmester, Angel Gómez, Alexandra Vázquez, William B. Swann

**Affiliations:** ^1^Department of Psychology, University of Texas at Austin, Austin, TX, United States; ^2^Institute of Cognitive and Evolutionary Anthropology, University of Oxford, Oxford, United Kingdom; ^3^Facultad de Psicología, Universidad Nacional de Educación a Distancia, Madrid, Spain; ^4^Artis International, Phoenix, AZ, United States

**Keywords:** identity fusion, sacred values, moral convictions, self-sacrifice, extremism, terrorism

## Abstract

Recent research has identified three promising candidates for predicting extreme behavior: sacred values, moral convictions, and identity fusion. Each construct is thought to motivate extreme behavior in unique ways: Sacred values trigger extreme actions when people are asked to compromise cause-related values for personal gain; moral convictions trigger extreme actions when a cause is aligned with one’s moral compass; and identity fusion triggers extreme actions when a cause is inextricably associated (“fused”) with the personal self. In six studies, we asked which of the three constructs (either alone or in combination) was most predictive of sacrifice for a cause. We measured all three constructs with respect to either of two causes: gun rights (Studies 1–3) or abortion rights (4–6). The outcome measure was endorsement of fighting and dying for the cause. Although all three constructs were significant predictors of the outcome measure when considered separately, identity fusion consistently emerged as the strongest predictor of endorsement of self-sacrifice when all three were considered simultaneously. This pattern occurred regardless of the target cause (gun or abortion rights), the participant’s position on the cause (i.e., pro-gun or anti-gun, pro-choice, or pro-life), or nationality (American vs. Spanish). Also, there was no evidence that the predictors interacted to predict the outcome measure. Finally, a manipulation that threatened the validity of the personal self strengthened the relationship between endorsement of self-sacrifice and both (a) identity fusion and (b) moral convictions. The latter finding suggests that threats to the validity of one’s self-views may amplify the extreme behaviors of true believers.

## Introduction

“The true believer is everywhere on the march, and both by converting and antagonizing he is shaping the world in his own image. And whether we are to line up with him or against him, it is well that we should know all we can concerning his nature and potentialities.” (Hoffer, 1951)

Although Hoffer wrote over a half century ago, the “nature and potentialities” of true believers are still dimly understood. For example, the reasons why true believers enact extreme behaviors for their favored causes remain mysterious. Fortunately, three relatively new variables – sacred values, moral convictions, and identity fusion – may help illuminate the processes that motivate true believers. In this report, we ask which of these variables – either alone or in combination with each other – best predicts endorsement of fighting and dying for a cause. We chose these variables because we suspected that they may share a common element – the personal self – which might moderate the impact of each of these variables on endorsement of extreme behavior. We begin with a brief description of each of these variables.

### Sacred Values, Moral Convictions, and Identity Fusion as Predictors of Extreme Behavior

Tetlock et al. (1996) and Tetlock (2003) introduced the sacred value construct to explain what happens when there is a clash between an individual’s religious and economic imperatives. They proposed that when the moral community deems a value sacred, members of the community are expected to strenuously resist the use of economic incentives to persuade them to abandon the value. Later authors (Atran and Ginges, 2015) removed the religious component from sacred values, contending that “although the term ‘sacred values’ intuitively denotes religious belief, … we use the term to refer to any preferences regarding objects, beliefs, or practices that people treat as both incompatible or nonfungible with profane issues or economic goods.”

The defining characteristic of sacred values is absolute and unequivocal adherence to the value. In fact, non-negotiability is so central to the sacred values construct that some investigators (e.g., Sheikh et al., 2016; Gómez et al., 2017; Vázquez et al., 2020) measure the construct using a single-item assessment of non-negotiability (operationalized as refusal to compromise a value in exchange for material benefits). Consistent with expectation, research has indicated that those who claim that a value is non-negotiable are more inclined to endorse extreme behaviors to defend that value, including even sacrificing their life, letting their family suffer, killing civilians, undertaking a suicide attack, and torturing women and children (Atran and Ginges, 2015; Gómez et al., 2017).

Moral convictions could also motivate true believers to make extreme sacrifices. These convictions are feelings regarding what is right and wrong that constitute core aspects of the personal self (Skitka et al., 2005, 2021). Moral convictions theoretically foster a principled obligation to act that, in turn, predicts intentions to enact actions that advance the cause (Sabucedo et al., 2018). Like sacred values, moral convictions are perceived to be objectively true and universally applicable (Skitka, 2014) and are associated with an unwillingness to compromise even in the face of competing desires or concerns (Skitka, 2014). For example, whereas a strong anti-abortion belief might rule out abortion under *most* circumstances, a moral conviction against abortion will rule out abortion under *all* circumstance*s* – even if, for example, it is certain that both the mother and fetus will die during childbirth.

Yet, moral convictions are distinct from sacred values in at least one respect. Whereas sacred values are theoretically dictated by the moral community, moral convictions are understood to be independent of establishment, convention, rules, or authorities (Skitka et al., 2008). As such, normative and majority considerations should have relatively little influence on moral convictions or associated obligations to act. For example, Americans who held a moral conviction against torture resisted a majority norm that supported the torture of suspected terrorists (Aramovich et al., 2012).

Identity fusion is a third variable that may motivate the extreme actions of true believers. Identity fusion occurs when an abstraction (a group, cause, or even another person) comes to define the self. When people become fused to a target group or cause, the boundaries between the self and the target become porous and the personal self becomes one with the target. This union creates a sense of equivalence of the self and the target that makes defending the target equivalent to defending the self (Swann et al., 2009, 2012). As a result, strongly fused persons are especially prone to enact pro-group or pro-cause behaviors when under threat from perceived adversaries (Swann et al., 2014; Fredman et al., 2017). The bulk of past research on identity fusion has emphasized the antecedents and consequences of identity fusion with groups (see, for example, Jong et al., 2015; Swann and Buhrmester, 2015; Gómez et al., 2020). Nevertheless, there is now work demonstrating the consequences of being fused with various causes, including religion (Fredman et al., 2017), political party (Buhrmester et al., 2012; Ashokkumar et al., 2019; Talaifar and Swann, 2019), gun and abortion rights (Ashokkumar et al., 2020), and even politicians, such as Donald Trump (e.g., Kunst et al., 2019; Martel et al., in preparation).

Although sacred values, moral convictions, and identity fusion have garnered considerable attention, efforts to integrate them have been limited. One reason for this may be that researchers have been mindful of important distinctions between these approaches. For example, whereas the sacred values and moral conviction formulations explicitly include a moral component, the identity fusion formulation includes no explicit moral component. Nevertheless, the identity fusion formulation may accommodate moral considerations because such considerations represent an aspect of the personal self for most people. For this reason, aligning the personal self with a target of fusion is tantamount to imbuing the target with moral overtones. From this vantage point, the identity fusion formulation is a broader construct that can readily accommodate material as well as moral beliefs (e.g., Carnes and Lickel, 2018; Chinchilla et al., 2021).

Methodological factors have also hampered efforts to assess the relationship between the three potential predictors of extreme behaviors of true believers. For example, the use of single-item measures of fusion and sacred values (Atran and Ginges, 2015) has precluded factor analytic assessments of the relationship between the two variables. In addition, past researchers have typically focused on one cause and sampled participants from one country. To address these limitations, in our research, we (a) used multi-item measures of each predictor, (b) tethered measures of the three potential predictors to either of two specific causes (abortion or gun rights), and (c) sampled participants from two countries (United States and Spain). The outcome measure was endorsement of fighting and dying for the cause under scrutiny. This allowed us to systematically assess the relationship between the predictors and compare the capacity of each to predict willingness to fight and die for a cause both alone and in interaction with one another.

### Is There a Common Mechanism Underlying the Effects of Sacred Values, Moral Convictions, and Identity Fusion?

Our research also asked why true believers care so deeply about sacred values, moral convictions, and identity fusion. Our search for answers to this question prompted us to consult theory and research on attitudes and behavior. This literature indicates that people appear to care most about beliefs that are highly important and central to the personal self (e.g., Petty and Krosnick, 1995). Hence, true believers may simply regard sacred values, moral convictions, and targets of fusion as particularly relevant to their personal selves. We tested this possibility in our research using a series of four manipulations, each designed to increase the salience of the personal self in a unique way. We reasoned that insofar as the personal self underlies the impact of a given predictor variable (i.e., sacred values, moral convictions, or identity fusion) on willingness to self-sacrifice for a cause, increasing the salience of the personal self would strengthen the relationship between that predictor variable and willingness to fight and die for the cause.

To select manipulations to increase the salience of the personal self, we drew upon the social psychological literature on self and identity. This literature pointed to two distinct approaches for increasing the salience of the personal self. The most common approach involves encouraging participants to affirm some aspect of the personal self. We considered three such self-affirmation manipulations. First, participants completed a series of 5 sentences, each of which began with “I am a” by responding with the first things that came to mind (Kuhn and McPartland, 1954). Second, participants imagined the most personal goals and dreams they have hoped to accomplish before their death as well as the legacy they hoped to leave behind (*cf*. Klackl and Jonas, 2019). Third, participants wrote about what makes them unique (Silvia and Eichstaedt, 2004), that is, “What makes you, ‘you?’”

As an alternative to the three self-affirmation manipulations, in our final study, we employed self-*dis*confirming feedback. The rationale underlying this manipulation comes from self-verification theory (Swann, 1983). Specifically, when people receive feedback from others that threatens aspects of their personal self, they may systematically work to refute the disconfirming feedback (e.g., Swann and Hill, 1982). Researchers have shown that self-disconfirming feedback increases the relation between identity fusion and endorsement of extreme behavior (Swann et al., 2009; Gómez et al., 2011).

## Overview of Our Research

As noted above, our studies focused on two different causes. Study cluster I (#1–3) focused on gun rights, and study cluster II (#4–6) focused on abortion rights. Also, the first study within each cluster (i.e., #1 and #4) included no manipulation of the personal self, which is to say only four of the six studies included such a manipulation (Studies #2–3, #5–6). Finally, Studies #1–5 recruited American participants through the Prolific crowdsourcing platform; Study 6 used a snowball technique facilitated by introductory psychology students from Spain.

We addressed four primary questions. First, what was the relationship of the three predictors to one another? Second, to what degree were each of the three predictors uniquely related to endorsement of extreme behavior? Third, were the predictors stronger when predicting the outcome variable on their own or in interaction with each other? Finally, with respect to the studies that had experimental manipulations (# 2, 3, 5, 6), did the manipulation interact with any of the three predictors in predicting endorsement of extreme behavior? We address each of these four questions in the research that follows.

## Study Cluster I: Sacred Values, Moral Convictions, and Identity Fusion as Predictors of Willingness to Self-Sacrifice for the Gun Rights Cause

### Study 1

#### Method

##### Participants

We recruited 311 American participants through Prolific. In this study and all subsequent studies, we excluded participants who failed attention checks, failed to complete the survey, or were outliers on the predictor or outcome variables. Outliers were identified by examining box plots of the variables and through the use of R’s “boxplot.stats” function. After exclusions, 291 participants remained (130 male, 157 female, 4 other; ages 18–73; 102 pro-gun, 189 anti-gun).

##### Procedure

All studies reported here shared a common core procedure which included introducing the study as an investigation of participants’ opinions toward a controversial contemporary issue. Participants then indicated whether they opposed or supported gun restrictions (Studies 1–3) or access to abortion (Studies 4–6). They then completed measures of the three target predictors (sacred values, moral convictions, and identity fusion). As Studies 1 and 4 had no experimental manipulation, participants completed the outcome measure (willingness to self-sacrifice for their position on the gun/abortion cause) immediately after competing measures of the three predictors. In Studies 2–3 and 5–6, participants received the experimental manipulation prior to completing the outcome measure.

##### Measures of Predictors and Outcome

In all 6 studies, participants completed, in random order, the measures of the three predictors (sacred values, moral convictions, and identity fusion). The outcome measure was always willingness to self-sacrifice for the cause. We describe these measures below and present the relevant descriptive statistics in SOM-1.

Predictor 1: Sacred Values. Our primary measure of sacred values was a continuous, 4-item measure adapted from Hanselmann and Tanner (2008). Participants indicated whether their stance on the gun rights issue was open to material trade-offs (e.g., “My position on gun control is something that I should not sacrifice, no matter what the benefits (money or something else)”; “My position on gun control is non-negotiable”). Participants indicated the degree to which they agreed with each statement on scales ranging from 1 (*completely disagree*) to 7 (*completely agree*). In our final two studies, we also assessed sacred values using a modified version of the single-item, dichotomous measure employed by Sheikh et al. (2016). Because the continuous measure was a stronger predictor than the dichotomous one, we present the results of the continuous predictor in the body of the paper and relegate the results of the dichotomous predictor to the SOM (see SOM-5).

Predictor 2: Moral Convictions. We used the 5-item measure of moral convictions (Morgan, 2012; Skitka, 2014) to measure the degree to which participants’ stance on the gun rights issue is related to their personal sense of morality (e.g., “To what extent do you feel your position on gun control is based on strong personal principles?”; “How much are your feelings about your position on gun control connected to your core moral beliefs and convictions?”). All items were measured on scales ranging from 1 (*not at all*) to 5 (*extremely*).

Predictor 3: Identity Fusion. Participants completed a measure of identity fusion with their position on the gun rights cause using a modified version of Gómez et al.’s (2011) seven-item continuous fusion scale (e.g., “I am strong because of my position on gun control”; “I am one with my position on gun control”). The respondents indicated the degree to which each statement reflected their relationship with the gun rights cause on scales ranging from 1 (*completely disagree*) to 7 (*completely agree*).

Outcome Measure: Willingness to Self-Sacrifice. We measured participants’ willingness to self-sacrifice in defense of their position on the gun rights cause with the 7-item scale developed by Swann et al. (2009). The items assessed willingness to fight or even die in defense of the cause (e.g., “I would fight someone threatening my position on gun control”; “I would sacrifice my life if it advanced my position on gun control”). On scales ranging from 1 (*completely disagree*) to 7 (*completely agree*), respondents indicated the degree to which each statement reflected their willingness to self-sacrifice for the gun control cause.

After responding to the outcome measure, participants then completed attention check items and demographic questions (see SOM-6). Finally, participants were debriefed.

Note: All R code and data files used for analyses are publicly available at OSF.^1^

#### Results

##### Covariation Among Predictors

As can be seen in Table 1, the correlations between the three predictors were moderate to substantial in most of the six studies (breaking samples down into participants who favored or opposed a given cause did not alter our conclusions).

**Table 1:** Correlations between predictors in all studies.

Study	Sacred values and moral convictions	Sacred values and identity fusion	Moral convictions and identity fusion
1	0.58	0.54	0.52
2	0.72	0.66	0.62
3	0.66	0.54	0.53
4	0.58	0.54	0.49
5	0.53	0.26	0.28
6	0.60	0.45	0.54

*For all correlations, ps<0.001*.

We also entered the three predictors into a series of factor analyses using oblimin rotation. With the exception of Study 6, the three predictors consistently loaded strongly to three unique factors (see Table 2 for an example). However, in Study 6, all items for the sacred values and moral convictions scales both loaded strongly on one factor, the first two identity fusion items loaded strongly on another factor, and the remaining five fusion items loaded on the final factor. The factor loadings for all six studies are presented in the SOM (SOM-2).

**Table 2:** Factor analyses loadings of predictors in Study 1.

Items	Factor 1	Factor 2	Factor 3
Fusion 1	**0.847**	0.138	
Fusion 2	**0.836**	0.142	0.164
Fusion 3	**0.765**	0.274	0.259
Fusion 4	**0.683**	0.267	0.297
Fusion 5	**0.686**	0.228	0.114
Fusion 6	**0.603**	0.156	0.233
Fusion 7	**0.628**	0.229	0.332
Sacred values 1	0.258	0.295	**0.652**
Sacred values 2	0.175	0.301	**0.556**
Sacred values 3	0.233	0.274	**0.907**
Sacred values 4	0.289	0.262	**0.788**
Moral convictions 1	0.241	**0.549**	0.220
Moral convictions 2	0.190	**0.734**	0.238
Moral convictions 3	0.131	**0.744**	0.234
Moral convictions 4	0.238	**0.767**	0.238
Moral convictions 5	0.254	**0.693**	0.213

*Here and in the SOM, blank spaces indicate that the factor loading value was very small (below absolute value of 0.1)*.

*Bold values indicate the strongest factor loading for each item*.

##### Predictive Validity of the Three Predictors

Analytic Approach and Statistical Notes Pertaining to All Studies. To determine whether sacred values, moral convictions, and identity fusion interactively predicted increased willingness to self-sacrifice for a cause, in each study, we tested for the 3-way interaction with a regression model that included the three-way interaction between the predictors, all two-way interactions, and all single predictors. To test for the 2-way interactions, we ran 3 unique models which contained each possible two-way interaction (fusion × sacred values, fusion × moral convictions, and sacred values × moral convictions) and the corresponding single predictors.

Next, to determine which predictor was the strongest predictor, we ran a simultaneous multiple regression model with sacred values, moral convictions, and identity fusion as predictors and self-sacrifice for a cause as the outcome. Finally, in the four studies which contained experimental manipulations, we ran regression models to test possible two-way interactions between each of the primary predictors with the experimental manipulation and then report any main effect of the manipulation alone. Here and hereafter, all regression models include the unstandardized beta coefficients, the unstandardized confidence intervals, the *t* test and associated value of *p* for the given effect, and the total model adjusted *R*^2^.

Let us add two important statistical notes. First, given the substantial correlations between the three predictors, we were concerned that multicollinearity could influence our findings. This concern was not supported. That is, in all six studies, the variance inflation factors never exceeded 2.50 (the specific values are presented in SOM-3). Second, to determine whether the three predictors were associated with the outcome measures when they were considered individually (i.e., without controlling for each other), we also ran single-predictor regressions (i.e., sacred values, moral convictions, and identity fusion) in which the outcome was willingness to self-sacrifice and the bivariate correlations between each predictor and willingness to self-sacrifice (see SOM-4). As shown in the Supplementary Material, sacred values and especially moral convictions were slightly more potent in single-predictor regressions than they were in the simultaneous multiple regressions. Sacred values were significant in Studies 1–4 and Study 6 (*p*s<0.05); moral convictions were significant in all six studies (*p*s<0.01) and identity fusion was as well (*p*s<0.001).

Analyses of Study 1. We first tested for the presence of triple- and two-way interactions between the three predictors (sacred values, moral convictions, and identity fusion). No significant two- nor three-way interactions between the three predictor variables emerged, *p*s>0.148.

Subsequent inspection of the main effects (with the interactions removed) revealed that identity fusion was the strongest predictor overall. That is, both identity fusion [*B*=0.18, 95% CI (0.11, 0.25), *t*(287)=4.94, *p*<0.001, total model $R_{\mathrm{adj}}^{2}$ =0.19] and sacred values [*B*=0.08, 95% CI (0.01, 0.16), *t*(287)=2.27, *p*=0.024] emerged as significant predictors. The difference between the effect size for fusion versus sacred values was marginally significant (*z*=1.85, *p*=0.06). Moral convictions (*p*=0.828) were not a significant predictor in this model.

### Study 2

#### Method

##### Participants

We recruited 122 American participants through Prolific. After exclusions, 108 (47 male, 58 female, 3 other; ages 18–79; 32 pro-gun, 76 anti-gun) remained.

##### Procedure

Participants first completed the three predictors. Then, in the self-affirmation condition, participants received a manipulation designed to increase the salience of the personal self. Specifically, participants responded to five statements that began “I am a ….” In the control condition, the five statements began, “Fish are ….” Then, on the following page, in both conditions participants were asked to write a brief explanation of the words they used to fill in the blanks. After the manipulation, participants completed the same outcome measure used in Study 1. Please see SOM-7 for the full text of all the manipulations.

#### Results

We first tested for the presence of triple- and two-way interactions between the three predictors (sacred values, moral convictions, and identity fusion). No significant two- nor three-way interactions between the three predictor variables emerged, *p*s>0.157.

Subsequent inspection of the main effects (with the interactions removed) revealed that identity fusion was the only significant predictor [*B*=0.37, 95% CI (0.21, 0.53), *t*(104)=4.68, *p*<0.001, total model $R_{\mathrm{adj}}^{2}$ =0.28]; neither sacred values (*p*=0.391) nor moral convictions (*p*=0.422) were significant.

Finally, there were no significant main nor interactive effects of the experimental manipulation on willingness to self-sacrifice for the cause (*p*s>0.269).

### Study 3

#### Method

##### Participants

For Study 3, we recruited 121 American participants through Prolific. After exclusions, 113 participants (45 male, 68 female; ages 18–70; 39 pro-gun, 74 anti-gun) remained.

##### Procedure

Participants completed the measures of the three predictors. Then, in the self-affirmation condition, participants received a manipulation designed to increase the salience of the personal self. Specifically, participants in the self-affirmation condition wrote about their goals prior to dying and the legacy they hoped to leave behind (“Please take a few minutes to write about what comes to mind when you think about your death. Please focus on (1) the most personal goals and dreams you’ll have hoped to accomplish before death and (2) the legacy that you hope to leave behind. Be as specific or general as you would like”). In the control condition, participants were asked to write about fish (“Please take a few minutes to write about fish and anything that comes to mind regarding them. Be as specific or general as you would like”). After responding to one of the two prompts, all participants then completed the outcome measure.

#### Results

We first tested for the presence of triple- and two-way interactions between the three predictors (sacred values, moral convictions, and identity fusion). No significant two- nor three-way interactions between the three predictor variables emerged, *p*s>0.418.

Subsequent inspection of the main effects (with the interactions removed) revealed that identity fusion was a marginally significant predictor of the outcome measure [*B*=0.14, 95% CI (−0.002, 0.29), *t*(109)=1.95, *p*=0.054, total model $R_{\mathrm{adj}}^{2}$ =0.14] but sacred values (*p*=0.466) and moral convictions (*p*=0.339) were not.

There were also no interactions between the manipulation and sacred values, moral conviction, or identity fusion in Study 3 (*p*s>0.549). Finally, there was no significant main effect of experimental manipulation on willingness to self-sacrifice for cause [*t*(111)=1.18, *p*=0.242].

### Summary of Findings From Cluster 1 Studies

Factor analytic results of our first three studies indicate that measures of sacred values, moral convictions, and identity fusion load onto separate factors. Moreover, when we compared the relative utility of the three variables in predicting willingness to sacrifice for the gun rights cause, identity fusion emerged as the strongest predictor, and there was no evidence of interactions between the three predictors. Finally, attempts to experimentally increase the salience of the personal self by affirming the personal self failed to increase endorsement of self-sacrifice for the cause.

## Study Cluster 2: Sacred Values, Moral Convictions, and Identity Fusion as Predictors of Willingness to Self-Sacrifice for the Abortion Rights Cause

Intrigued by these findings, we conducted three follow-up investigations. One goal was to determine whether the findings from study cluster I would generalize to an unrelated cause, abortion rights, and to a new sample, Spaniards. In addition, to determine whether self-confirming versus self-disconfirming manipulations would differentially influence the relationship between sacred values, moral convictions, or identity fusion and willingness to self-sacrifice, we introduced appropriate manipulations in Studies 5 and 6, respectively.

### Study 4

#### Method

##### Participants

We recruited 303 American participants through Prolific, 275 of which remained after exclusions (116 male, 152 female, 7 other; ages 18–72; 56 pro-life, 219 pro-choice).

##### Procedure

There was no experimental manipulation; instead, participants proceeded directly to the outcome measure after completing measures of the three predictors. Finally, in all studies, participants completed attention check items and demographic questions and then were debriefed.

#### Results

We first tested for the presence of triple- and two-way interactions between the three predictors (sacred values, moral convictions, and identity fusion). No significant two- nor three-way interactions between the three predictor variables emerged, *p*s>0.161.

Subsequent inspection of the main effects (with the interactions removed) revealed that identity fusion was the only significant predictor of willingness to self-sacrifice [*B*=0.29, 95% CI (0.20, 0.39), *t*(271)=6.10, *p*<0.001, total model $R_{\mathrm{adj}}^{2}$ =0.20]; sacred values (*p*=0.838) and moral convictions (*p*=0.328) were not significant.

### Study 5

#### Method

##### Participants

We recruited 342 American participants through Prolific. After exclusions, 288 remained (152 male, 133 female, 3 other; ages 18–64; 288 pro-choice). In this study, we only recruited pro-choice participants due to their greater availability and the fact that there were no apparent differences between pro-choice and pro-life participants in the foregoing study.

##### Procedure

Participants first completed measures of the three predictors. Then, in the self-affirmation condition, participants received a manipulation designed to increase the salience of the personal self. Specifically, participants imagined that they were describing their inner selves to a close friend (“Please take 2min to tell us about yourself. Imagine yourself with your closest friend and your friend asks you “What makes you ‘you?’” Imagine your friend isn’t interested in superficial qualities and really wants to know about your enduring, deepest self”). In the control condition, participants contemplated the existence of alien life (“Please take 2min to give your opinion about whether there is intelligent life in the universe other than on Earth”). Participants then completed the outcome measure.

#### Results

We first tested for the presence of triple- and two-way interactions between the three predictors (sacred values, moral convictions, and identity fusion). No significant two- nor three-way interactions between the three predictor variables emerged, *p*s>0.253.

Subsequent inspection of the main effects (with the interactions removed) revealed that identity fusion was a significant predictor [*B*=0.35, 95% CI (0.26, 0.44), *t*(284)=7.75, *p*<0.001, total model $R_{\mathrm{adj}}^{2}$ =0.22] and so too was moral convictions [*B*=0.24, 95% CI (0.08, 0.39), *t*(284)=3.05, *p*=0.003], but not sacred values (*p*=0.066). The significant effect of moral convictions in Study 5 was an exception to the overall pattern reported in this paper, but note that even so the fusion effect was stronger than the moral convictions effect (*z*=3.16, *p*<0.001).

There were no interactive effects of the manipulation and sacred values, moral conviction, or identity fusion in Study 5 (*p*s>0.491), nor was there a main effect of the manipulation (*p*=0.624).

### Study 6

#### Method

In contrast to the first five studies, in this study, we attempted to threaten the personal self by presenting participants with feedback that threatened their self-views, a manipulation which has been used in previous research to effectively activate the personal self (Swann et al., 2009; Gómez et al., 2011). To enhance the plausibility of the feedback manipulation, this study was conducted in two waves. Specifically, during wave one, participants completed some questionnaires. We ostensibly showed their responses to a team of psychologist evaluators prior to wave two, thus providing a basis for the feedback manipulation.

##### Participants

We recruited participants using the snowball technique wherein Spanish Psychology undergraduates asked their acquaintances to participate. Participation was voluntary and uncompensated. We recruited 267 Spanish participants in the first wave; 199 participants completed both waves, and 197 of these participants remained after exclusions and were included in our analyses (42 male, 155 female; ages 20–68; 19 pro-life, 178 pro-choice).

##### Procedure

In wave one, we measured the three predictors (sacred values, moral convictions, and identity fusion) with respect to the abortion cause. 1week later, participants received an email inviting them to complete wave two of the study, to which they responded within 1 to 39days. In wave two, we introduced the feedback manipulation. Participants learned that, based on their responses during wave one, they had been evaluated by a group of psychologists who had assessed how the participant perceived him/herself as well as how the participant actually is on five dimensions: shyness, insecurity, stubbornness, nervousness, and distrust. Participants in the self-disconfirming condition learned that the psychologists had concluded that, for four of the five dimensions, there was a discrepancy between participants’ self-views and their actual characteristics. In contrast, participants in the verifying condition learned that the psychologists had concluded that, for four of the five dimensions, their self-views agreed with their actual characteristics. Participants in the control condition learned that due to a technical problem, they would not receive any feedback from the evaluators. After the feedback manipulation, participants completed the outcome measure, willingness to self-sacrifice for the abortion cause.

#### Results

We first tested for the presence of triple- and two-way interactions between the three predictors (sacred values, moral convictions, and identity fusion). No significant two- nor three-way interactions between the three predictor variables emerged, *p*s>0.479.

Subsequent inspection of the main effects (with the interactions removed) revealed that identity fusion was a significant predictor [*B*=0.24, 95% CI (0.14, 0.35), *t*(193)=4.51, *p*<0.001, total model $R_{\mathrm{adj}}^{2}$ =0.12], but the other two predictors were not, sacred values (*p*=0.905), moral convictions (*p*=0.879).

**Figure 1:**
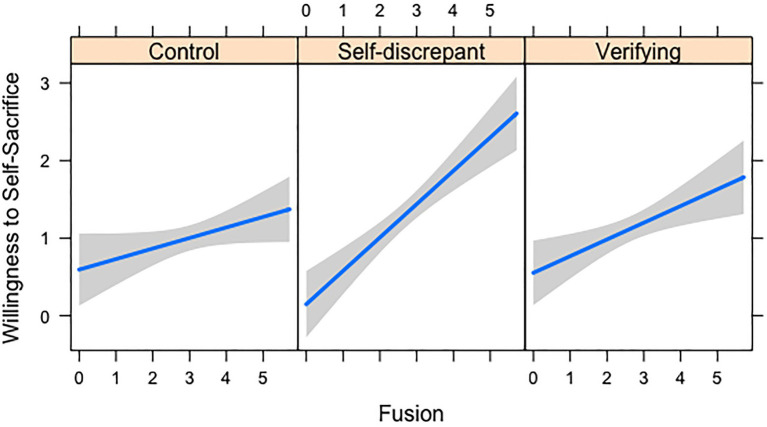
Study 6 interaction between fusion and experimental manipulation in predicting willingness to self-sacrifice.

We then tested whether each of the three primary predictors interacted with the experimental manipulation in three separate regression models in which we dummy-coded the self-disconfirming and verifying condition against the baseline control condition. When we regressed willingness to self-sacrifice for the cause on one of the three primary predictors, the two dummy-coded variables, and the two interaction terms between the primary predictor and the dummy-coded variables, a significant interaction emerged between the experimental manipulation and identity fusion. As shown in Figure 1, identity fusion was more strongly predictive of willingness to self-sacrifice in the self-disconfirming condition compared to the control condition [*B*=0.29, 95% CI (0.09, 0.50), *t*(191)=2.82, *p*=0.005, total model $R_{\mathrm{adj}}^{2}$ =0.19], whereas the predictive power of identity fusion did not differ between the verifying and control conditions [*B*=0.08, 95% CI (−0.13, 0.28), *t*(191)=0.77, *p*=0.444]. Simple effects analyses of the results displayed in Figure 1 indicated that fusion with abortion was a stronger predictor of willingness to self-sacrifice for the cause in the self-disconfirming condition [*B*=0.43, *t*(191)=5.79, *p*<0.001] than in the verifying condition [*B*=0.22, *t*(191)=2.93, *p*=0.004] or the control condition [*B*=0.14, *t*(191)=1.86, *p*=0.064].

**Figure 2:**
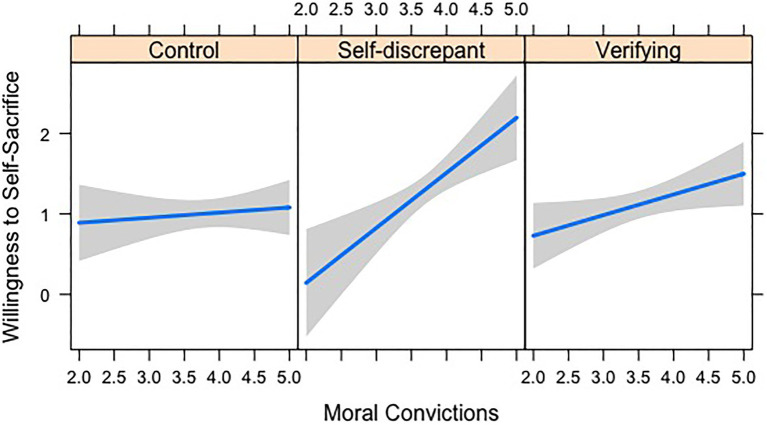
Study 6 interaction between moral convictions and experimental manipulation in predicting willingness to self-sacrifice.

There was also a significant interaction between moral convictions and the experimental manipulation. As shown in Figure 2, moral convictions were significantly more strongly predictive of willingness to self-sacrifice in the self-disconfirming condition compared to the control condition [*B*=0.62, 95% CI (0.17, 1.07), *t*(191)=2.71, *p*=0.007, total model $R_{\mathrm{adj}}^{2}$ =0.08], whereas the predictive power of moral convictions did not differ between the verifying and control conditions [*B*=0.19, 95% CI (−0.15, 0.54), *t*(191)=1.11, *p*=0.270]. Simple effects analyses of the results displayed in Figure 2 indicated that holding moral convictions toward one’s position on the abortion cause was a stronger predictor of willingness to self-sacrifice for the cause in the self-disconfirming condition [*B*=0.68, *t*(191)=3.55, *p*<0.001] than in the verifying condition [*B*=0.26, *t*(191)=2.10, *p*=0.037] or the control condition [*B*=0.06, *t*(191)=0.51, *p*=0.611].

In contrast, sacred values were not a significantly stronger predictor of willingness to self-sacrifice for the cause in the self-disconfirming condition compared to the control condition (*p*=0.999) or in the verifying condition compared to the control condition (*p*=0.498).

Finally, the significant interactions discussed above qualified a marginal main effect of the experimental manipulation on sacrifice for the cause [*F*(2,194)=2.61, *p*=0.076, *η*^2^=0.03]. This marginal main effect of *η*^2^=0.03 could be considered small (*η*^2^=0.01) to medium (*η*^2^=0.06) based on conventional interpretations of eta squared effect sizes (Cohen, 1988).

## General Discussion

If it is clear that true believers are movers and shakers who shape the future of the world, it is less clear what drives them to behave as they do. We attempted to address this gap in the literature by determining whether three variables – sacred values, moral convictions, and identity fusion – might contribute to the extreme behaviors of true believers. The results of six studies supported some, but not all, of our expectations. As anticipated, our findings consistently showed that although measures of the three constructs were correlated, they loaded onto separate factors. This suggests that the three predictors are related but distinct. Further support for this conclusion emerged when we entered the three predictors into simultaneous multiple regressions in which the outcome was endorsement of fighting and dying for a cause. The results of these regressions indicated that when we controlled for the effects of the other variables, identity fusion emerged as the strongest predictor.

Why was identity fusion a stronger predictor of self-sacrifice than either sacred values or moral convictions? We originally hypothesized that the predictive power of identity fusion stems from its sensitivity to the degree to which the personal self is aligned with the target of fusion. Contrary to this hypothesis, affirming the personal self in Studies 2, 3, and 5 did not strengthen the relationship between fusion and endorsement of extreme behavior for the cause.

Nevertheless, in Study 6, providing participants with self-disconfirming feedback interacted with identity fusion such that highly fused participants were particularly inclined to endorse extreme behavior and weakly fused participants were particularly disinclined to endorse extreme behavior. Perhaps disconfirming the self is a particularly effective way of activating the personal self. Alternatively, or in addition, having several experts disconfirm one’s self-views may represent a potent threat that compels actions designed to neutralize perceived threats.

Another approach to understanding the power of fusion to predict willingness to self-sacrifice for a cause is to consider why its rivals were relatively weak predictors. Consider sacred values. Whereas indices of identity fusion are framed in terms of positive sentiments (e.g., “I have a deep emotional bond with my position on gun control,” “Gun control is me”), indices of sacred values are framed in terms of negative sentiments (e.g., “My position on gun control is something that I should not sacrifice, no matter what the benefits (money or something else),” “My position on gun control is non-negotiable”). The negative framing of the sacred values items may be less motivating than the positive framing of the fusion items. A related possibility is that measures of sacred values focus on moral prohibitions against “selling out” (i.e., abdicating one’s values for material gain). Given that people are terrible at estimating their ability to resist social pressures (e.g., Milgram, 1963), answers to questions about selling out may be inherently unreliable. In any event, the value of positive framing might explain the success of measures of sacred values in predicting costly self-sacrifices on the battlefield in Iraq, as in that context sacred values are framed as a component of the fighters’ battle cry (Gómez et al., 2017). An alternative explanation for the anemic performance of sacred values in our studies is that sacred values are particularly influential in the context of intergroup conflicts (e.g., Sheikh et al., 2012), and such conflicts were not emphasized in our studies.

Like sacred values, moral convictions were a weaker predictor of endorsing self-sacrifice for a cause than identity fusion. Even so, moral convictions were a stronger predictor of self-sacrifice than sacred values. One reason for this is suggested by the results of Study 6. In that study, self-disconfirming feedback strengthened the relation between endorsement of self-sacrifice and both moral convictions and fusion (but not sacred values). Future research could seek to identify the mechanisms underlying these findings.

## Limitations, Implications, and Related Formulations

The results of our studies indicate that all three of the constructs we focused on here (sacred values, moral convictions, and identity fusion) were correlated with endorsement of fighting and dying for a cause. This suggests that measures of all three constructs could be used to identify potential true believers. That said, our simultaneous multiple regressions indicated that identity fusion was the most powerful predictor of endorsement of extreme behavior in our studies. Hence, it may be that researchers interested in extreme behavior will get more “bang for their buck” if they measure fusion rather than sacred values or moral convictions.

Of course, it may be that measures of sacred values or moral convictions would have been more effective if we had examined alignment with groups, other causes or if we had focused on different outcome measures. Moreover, even if our measure of identity fusion were generally superior to the measures of the rival constructs, this could say more about the measures themselves rather than the constructs they were designed to measure. For example, it could be that our measure of identity fusion is psychometrically superior to the particular measures of sacred values and moral convictions but that more reliable or valid measures of these rival variables would out-predict the identity fusion measure. Future research should explore these possibilities.

The six online surveys reported here provided consistent evidence that identity fusion, sacred values, and moral convictions all positively predicted stated willingness to fight and die for a cause. Whether and how support for such extreme actions would translate into actual behavior is beyond the scope of these studies. That said, field research conducted during the 2011 Libyan civil war indicated that fusion with one’s battalion was associated with whether militiamen volunteered to fight on the front lines rather than provide logistical support (Whitehouse et al., 2014). Other recent research conducted in prisons indicated that fusion with religion is associated with costly sacrifices for religion among inmates incarcerated because of Islamist terrorism (Gómez et al., 2021). The results of these studies thus provide some evidence that identity fusion is related to behavior in naturally occurring settings.

Of relevance to the true believer theme with which we opened this article, our findings suggest that people who are strongly fused with a cause may sometimes constitute “radicals-in-waiting,” especially if their cherished cause or their personal identity is threatened. Of course, whether highly fused persons actually radicalize depends on the target of their fusion; individuals who are strongly fused with radical jihadists are much more likely to fight and die for their group than those who are strongly fused with a rock band.

If being fused with certain groups or ideologies makes individuals potential radicals, then it makes sense to build comprehensive models of the variables that may prompt highly fused people to translate their feelings of fusion into violent action. The devoted actor model (Atran and Ginges, 2015), which combines identity fusion with sacred values, represents one such model (although our findings offered little evidence for the unique predictive utility of sacred values). Another candidate is the 3N model (e.g., Webber and Kruglanski, 2017; Bélanger et al., 2018, 2019), which examines the influence of needs, narratives, and social networks on radicalization. Due to its expansiveness, the 3N model provides a relatively comprehensive model of the variables that may motivate true believers to translate their convictions into extreme behavior.

Our evidence in Study 6 that a threat to the personal self amplified the effect of identity fusion is consistent with the 3N model’s emphasis on the importance of the desire for personal significance. It is also reminiscent of Hoffer’s (1951) comments on the role of perceived threat among true believers: “A rising mass movement attracts and holds a following not by its doctrine and promises but by the refuge it offers from the anxieties, barrenness and meaninglessness of an individual existence …” (Hoffer, 1951). Through their identity fusion with a cause, true believers may feel the self and the target of fusion to be functionally equivalent, which makes defending the target equivalent to defending the self (Swann et al., 2009, 2012).

Overall, we uncovered consistent evidence that identity fusion was the strongest predictor of willingness to fight and die regardless of participants’ position regarding abortion or gun rights. That said, the fact that our sample in Study 6 was predominantly composed of pro-choice, participants (178 pro-choice, 19 pro-life) raises the possibility that the results of this particular study were primarily driven by pro-choice participants.

Although our discussion thus far has focused on the dangers that true believers pose to the world at large, it is important to acknowledge that the degree of threat posed by true believers depends largely on the nature of the cause to which they are fused. In fact, identity fusion is socially beneficial in some instances. For example, students who were fused to their universities were more inclined to persist in college (Talaifar et al., 2021).

These caveats notwithstanding, when true believers become fused with terrorists or violent insurgents, it is important to develop effective intervention strategies (e.g., Kruglanski et al., 2014). Our findings suggest that the road to deradicalization will be a steep and thorny one for those who become fused with a cause because, for such individuals, deradicalization will mean relinquishing an aspect of their personal self. One strategy for managing the zealotry of true believers is to re-direct their passions from destruction (e.g., terrorism) to construction (e.g., building community). Alternatively, it may be possible to diminish identity fusion by degrading relational ties to other advocates of the group or cause (Gómez et al., 2019). In the latter case, focusing on disengagement from the group could be more effective than de-radicalization, as the latter requires surmounting the high bar of de-sacralization or de-fusion with a cause. Although the most effective way of dealing with true believers gone bad is not yet apparent, it is clear that achieving this goal is vitally important. Rather than attempting to bring true believers to disbelieve, it may be more realistic to bring them to believe in something else.

## Data Availability Statement

The datasets presented in this study can be found in online repositories. The names of the repository/repositories and accession number(s) can be found at: https://osf.io/p58ks/?view_only=1e250b9e2ff84465a0d9cffee89260ae.

## Ethics Statement

The studies involving human participants were reviewed and approved by The Institutional Review Board (IRB) at the University of Texas at Austin. The patients/participants provided their written informed consent to participate in this study.

## Author Contributions

FM, MB and WS were principally responsible for the design of the studies and FM and WS wrote the manuscript. FM collected the data for studies 1–5, wrote the R code and conducted analyses for all studies. AG and AV were principally responsible for the design and data collection of Study 6 and contributed to the analysis and editing of the manuscript. All authors contributed to the article and approved the submitted version.

## Funding

This research was supported by a National Science Foundation (#1761238) grant to WS, grants from the Spanish Ministry of Science, Innovation and Universities (#RTI2018-093550-B-I00) and AFOSR (#FA9550-17-C-0023 P00003) to AG, and a grant from the Spanish Ministry of Science, Innovation and Universities (#s RTI2018-098576-A-I00) to AV.

## Conflict of Interest

The authors declare that the research was conducted in the absence of any commercial or financial relationships that could be construed as a potential conflict of interest.

## Publisher’s Note

All claims expressed in this article are solely those of the authors and do not necessarily represent those of their affiliated organizations, or those of the publisher, the editors and the reviewers. Any product that may be evaluated in this article, or claim that may be made by its manufacturer, is not guaranteed or endorsed by the publisher.

## Footnotes

^1^ https://osf.io/p58ks/?view_only=1e250b9e2ff84465a0d9cffee89260ae
