# Supplementary material for: Why True Believers Make the Ultimate Sacrifice: Sacred Values, Moral Convictions, or Identity Fusion?
Source: Front Psychol. 2021 Nov 15;12:779120. doi: 10.3389/fpsyg.2021.779120 (PMC8634031; doi:10.3389/fpsyg.2021.779120)
Supplement: Supplementary file 1 [file Table_1.DOCX]

**Supplemental Online Materials**

**SOM-1 Descriptive Statistics**

*Supplementary Table 1, Fusion Descriptive Stats*

| Study # | Means | Standard Deviations | Cronbach Alphas |
| --- | --- | --- | --- |
| 1 | 3.89 | 1.47 | 0.92 |
| 2 | 3.88 | 1.42 | 0.92 |
| 3 | 3.77 | 1.60 | 0.94 |
| 4 | 4.46 | 1.44 | 0.91 |
| 5 | 3.95 | 1.28 | 0.88 |
| 6 | 2.78 | 1.15 | 0.84 |

*Supplementary Table 2, Sacred Values Descriptive Stats*

| Study # | Means | Standard Deviations | Cronbach Alphas |
| --- | --- | --- | --- |
| 1 | 5.09 | 1.53 | 0.90 |
| 2 | 4.81 | 1.51 | 0.90 |
| 3 | 5.10 | 1.52 | 0.89 |
| 4 | 5.94 | 1.20 | 0.89 |
| 5 | 5.52 | 1.26 | 0.80 |
| 6 | 4.08 | 1.44 | 0.81 |

*Supplementary Table 3, Moral Convictions Descriptive Stats*

| Study # | Means | Standard Deviations | Cronbach Alphas |
| --- | --- | --- | --- |
| 1 | 3.78 | 0.88 | 0.87 |
| 2 | 3.68 | 1.03 | 0.91 |
| 3 | 3.72 | 1.00 | 0.91 |
| 4 | 4.12 | 0.82 | 0.86 |
| 5 | 3.94 | 0.85 | 0.81 |
| 6 | 3.67 | 0.66 | 0.72 |

*Supplementary Table 4, Self-Sacrifice for Cause Descriptive Stats*

| Study # | Means | Standard Deviations | Cronbach Alphas |
| --- | --- | --- | --- |
| 1 | 1.87 | 0.81 | 0.81 |
| 2 | 2.05 | 0.99 | 0.86 |
| 3 | 1.96 | 0.91 | 0.83 |
| 4 | 2.28 | 1.04 | 0.84 |
| 5 | 2.13 | 1.05 | 0.86 |
| 6 | 1.14 | 0.76 | 0.76 |

**SOM-2 Factor Analysis Loadings**

As can be seen when examining the factor loadings for a three-factor model, each of the three constructs strongly maps to its own unique factor, with the exception of Study 6. The highest factor loadings for each item are bolded in the following tables.

*Supplementary Table 5, Study 1 Factor Loadings*

| Items | Factor 1 | Factor 2 | Factor 3 |
| --- | --- | --- | --- |
| Fusion1 | **0.847** | 0.138 |  |
| Fusion2 | **0.836** | 0.142 | 0.164 |
| Fusion3 | **0.765** | 0.274 | 0.259 |
| Fusion4 | **0.683** | 0.267 | 0.297 |
| Fusion5 | **0.686** | 0.228 | 0.114 |
| Fusion6 | **0.603** | 0.156 | 0.233 |
| Fusion7 | **0.628** | 0.229 | 0.332 |
| SacredValues1 | 0.258 | 0.295 | **0.652** |
| SacredValues2 | 0.175 | 0.301 | **0.556** |
| SacredValues3 | 0.233 | 0.274 | **0.907** |
| SacredValues4 | 0.289 | 0.262 | **0.788** |
| MoralConvictions1 | 0.241 | **0.549** | 0.220 |
| MoralConvictions2 | 0.190 | **0.734** | 0.238 |
| MoralConvictions3 | 0.131 | **0.744** | 0.234 |
| MoralConvictions4 | 0.238 | **0.767** | 0.238 |
| MoralConvictions5 | 0.254 | **0.693** | 0.213 |

*Supplementary Table 6, Study 2 Factor Loadings*

| Items | Factor 1 | Factor 2 | Factor 3 |
| --- | --- | --- | --- |
| Fusion1 | **0.620** |  | 0.326 |
| Fusion2 | **0.706** | 0.149 | 0.247 |
| Fusion3 | **0.789** | 0.266 | 0.185 |
| Fusion4 | **0.776** | 0.364 | 0.242 |
| Fusion5 | **0.725** | 0.308 | 0.119 |
| Fusion6 | **0.680** | 0.247 | 0.175 |
| Fusion7 | **0.678** | 0.301 | 0.310 |
| SacredValues1 | 0.364 | 0.441 | **0.538** |
| SacredValues2 | 0.173 | 0.331 | **0.612** |
| SacredValues3 | 0.377 | 0.351 | **0.821** |
| SacredValues4 | 0.395 | 0.339 | **0.670** |
| MoralConvictions1 | 0.251 | **0.550** | 0.416 |
| MoralConvictions2 | 0.255 | **0.746** | 0.178 |
| MoralConvictions3 | 0.256 | **0.692** | 0.323 |
| MoralConvictions4 | 0.299 | **0.813** | 0.303 |
| MoralConvictions5 | 0.263 | **0.799** | 0.301 |

*Supplementary Table 7, Study 3 Factor Loadings*

| Items | Factor 1 | Factor 2 | Factor 3 |
| --- | --- | --- | --- |
| Fusion1 | **0.873** | 0.175 | 0.259 |
| Fusion2 | **0.873** | 0.204 | 0.198 |
| Fusion3 | **0.766** | 0.257 | 0.334 |
| Fusion4 | **0.611** | 0.294 | 0.490 |
| Fusion5 | **0.713** | 0.326 | 0.244 |
| Fusion6 | **0.600** | 0.317 | 0.305 |
| Fusion7 | **0.563** | 0.324 | 0.335 |
| SacredValues1 | 0.328 | 0.246 | **0.631** |
| SacredValues2 | 0.268 | 0.433 | **0.464** |
| SacredValues3 | 0.278 | 0.306 | **0.844** |
| SacredValues4 | 0.453 | 0.217 | **0.739** |
| MoralConvictions1 | 0.216 | **0.654** | 0.234 |
| MoralConvictions2 | 0.140 | **0.843** | 0.207 |
| MoralConvictions3 | 0.260 | **0.714** | 0.153 |
| MoralConvictions4 | 0.307 | **0.813** | 0.239 |
| MoralConvictions5 | 0.248 | **0.755** | 0.214 |

*Supplementary Table 8, Study 4 Factor Loadings*

| Items | Factor 1 | Factor 2 | Factor 3 |
| --- | --- | --- | --- |
| Fusion1 | **0.718** | 0.194 | 0.153 |
| Fusion2 | **0.725** | 0.245 | 0.209 |
| Fusion3 | **0.796** | 0.191 | 0.277 |
| Fusion4 | **0.731** | 0.205 | 0.296 |
| Fusion5 | **0.804** | 0.158 | 0.112 |
| Fusion6 | **0.683** | 0.110 |  |
| Fusion7 | **0.605** | 0.228 | 0.337 |
| SacredValues1 | 0.233 | 0.279 | **0.617** |
| SacredValues2 | 0.215 | 0.288 | **0.618** |
| SacredValues3 | 0.232 | 0.233 | **0.870** |
| SacredValues4 | 0.258 | 0.269 | **0.771** |
| MoralConvictions1 | 0.228 | **0.616** | 0.316 |
| MoralConvictions2 | 0.160 | **0.740** | 0.137 |
| MoralConvictions3 | 0.188 | **0.664** | 0.327 |
| MoralConvictions4 | 0.195 | **0.694** | 0.301 |
| MoralConvictions5 | 0.209 | **0.682** | 0.146 |

*Supplementary Table 9, Study 5 Factor Loadings*

| Items | Factor 1 | Factor 2 | Factor 3 |
| --- | --- | --- | --- |
| Fusion1 | **0.597** |  |  |
| Fusion2 | **0.723** | 0.122 | 0.144 |
| Fusion3 | **0.857** |  | 0.114 |
| Fusion4 | **0.809** | 0.207 | 0.194 |
| Fusion5 | **0.729** | 0.107 |  |
| Fusion6 | **0.580** |  |  |
| Fusion7 | **0.602** | 0.123 |  |
| SacredValues1 |  | 0.271 | **0.809** |
| SacredValues2 | 0.113 | 0.123 | **0.797** |
| SacredValues3 | 0.175 | 0.463 | **0.545** |
| SacredValues4 |  | 0.278 | **0.461** |
| MoralConvictions1 | 0.107 | **0.557** | 0.329 |
| MoralConvictions2 |  | **0.684** | 0.107 |
| MoralConvictions3 |  | **0.590** | 0.256 |
| MoralConvictions4 | 0.136 | **0.741** | 0.205 |
| MoralConvictions5 | 0.185 | **0.688** |  |

*Supplementary Table 10, Study 6 Factor Loadings*

| Items | Factor 1 | Factor 2 | Factor 3 |
| --- | --- | --- | --- |
| Fusion1 | 0.141 | 0.252 | **0.822** |
| Fusion2 | 0.192 | 0.161 | **0.965** |
| Fusion3 | 0.332 | **0.388** | 0.248 |
| Fusion4 | 0.397 | **0.468** | 0.150 |
| Fusion5 | 0.164 | **0.758** | 0.192 |
| Fusion6 |  | **0.821** | 0.170 |
| Fusion7 | 0.230 | **0.761** |  |
| SacredValues1 | **0.710** | 0.167 |  |
| SacredValues2 | **0.531** |  |  |
| SacredValues3 | **0.761** | 0.171 | 0.123 |
| SacredValues4 | **0.719** | 0.260 | 0.165 |
| MoralConvictions1 | **0.450** | 0.263 | 0.133 |
| MoralConvictions2 | **0.426** | 0.199 | 0.144 |
| MoralConvictions3 | **0.560** |  | 0.171 |
| MoralConvictions4 | **0.373** | 0.339 | 0.265 |
| MoralConvictions5 | **0.439** | 0.151 |  |

Note that Study 6 was the one exception to the pattern, in that each of the three constructs did not load strongly and uniquely to three separate factors. In this case, Sacred Values and Moral Convictions both loaded strongly to the same factor (Factor 1) whereas as Identity Fusion loaded strongly to two unique factors (Factors 2 and 3).

**SOM-3 Variance inflation factor of multiple predictor models**

To ensure that the three primary constructs did not have problematic high levels of multicollinearity in the multiple predictor models, we ran the VIFs (Variance Inflation Factor) for each study.

*Supplementary Table 11, Variance in Inflation Factors (VIF)*

| Study # | Fusion | Sacred Values | Moral Convictions |
| --- | --- | --- | --- |
| 1 | 1.55 | 1.70 | 1.65 |
| 2 | 1.92 | 2.50 | 2.25 |
| 3 | 2.20 | 2.20 | 1.71 |
| 4 | 1.51 | 1.71 | 1.61 |
| 5 | 1.10 | 1.41 | 1.43 |
| 6 | 1.48 | 1.55 | 1.73 |

**SOM-4 Single Predictor Models and Bivariate Correlations between Predictors and Outcome**

*Supplementary Table 12, Main effects on sacrifice for the cause in single predictor models*

| Study | Identity Fusion | Sacred Values | Moral Convictions |
| --- | --- | --- | --- |
| 1 | 0.23*** | 0.18*** | 0.25*** |
| 2 | 0.38*** | 0.26*** | 0.30** |
| 3 | 0.22*** | 0.20*** | 0.29*** |
| 4 | 0.32*** | 0.24*** | 0.35*** |
| 5 | 0.37*** | 0.08, *p = 0.110* | 0.31*** |
| 6 | 0.24*** | 0.09* | 0.23** |

*** = *p* < 0.001, ** = *p* < 0.01, * = *p* < 0.05

*Supplementary Table 13, Bivariate correlations between predictors and the outcome measure*

| Study | Sacred Values and Willingness to Self-Sacrifice for Cause | Moral Convictions and Willingness to Self-Sacrifice for Cause | Identity Fusion and Willingness to Self-Sacrifice for Cause |
| --- | --- | --- | --- |
| 1 | 0.34 | 0.27 | 0.42 |
| 2 | 0.39 | 0.31, *p* = 0.001 | 0.54 |
| 3 | 0.35 | 0.30 | 0.46 |
| 4 | 0.27 | 0.28 | 0.45 |
| 5 | 0.09, *p* = 0.110 | 0.25 | 0.45 |
| 6 | 0.16, *p* = 0.021 | 0.21, *p* = 0.004 | 0.37 |

Note: For all correlations, *p’s* < 0.001 unless otherwise stated

**SOM-5 Analyses with Single-Item Measure of Sacred Values**

In the final two studies we also included a single item measure of sacred values. This measure was coded as a binary value, with participants who indicated that they would compromise their value for money (values 1-4 on the scale) coded as 0 for not holding it as a sacred value; participants who indicated that they would not compromise their value for any amount of money (value 5 on the scale) were coded as 1 for holding it as a sacred value. The full text of the measure is given below, as well as analyses from each study where it was present.

1-item Sacred Values Measure:

“How much money would be necessary for you to say you give up your actual position on abortion? (you can keep that money or donate it)”

🡪 0-- 0$, 1-- $100, 2-- $1000, 3-- 100,000), 4-- 100,000,000, 5 -- Never. The quantity does not matter

In Studies 5 and 6 which included this single-item sacred values measure, we ran single predictor models with it predicting willingness to self-sacrifice for cause; we also included it in multiple predictor regressions with identity fusion and moral convictions where we substituted this 1-item sacred values measure for the 4-item sacred values measure that we used in all studies. Finally, we ran interaction models to see if this 1-item measure interacted with the other main predictors, fusion and moral convictions, as well as whether this measure interacted with the experimental manipulation. Results are given below.

Study 5: The single-item sacred values item did not predict willingness to sacrifice for cause in a single predictor model (*p* = 0.295). It also did not significantly predict the same outcome in a multiple predictor model (*p* = 0.557) with both identity fusion and moral convictions entered as simultaneous predictors. Finally, the single-item sacred values item did not interact with the experimental manipulation (*p* = 0.587), nor did it interaction with fusion (*p* = 0.932) or moral convictions (*p* = 0.723).

Study 6: The single-item sacred values item did not predict willingness to sacrifice for cause in a single predictor model (*p* = 0.422). It also did not significantly predict the same outcome in a multiple predictor model with both identity fusion and moral convictions entered as simultaneous predictors (*p* = 0.591). Finally, the single-item sacred values item did not interact with the experimental manipulation conditions in which we dummy coded the self-disconfirming (*p* = 0.705) condition and the verifying condition (*p* = 0.419) against the baseline control condition, nor did it interaction with fusion (*p* = 0.851) or moral convictions (*p* = 0.434).

**SOM-6 Full Text of Measures**

Predictor Measures

Identity Fusion (scale from 0 (*Completely Disagree*) to 6 (*Completely Agree*); coded as values between 1-7)

1. My position on [abortion/gun control] is me.
2. I am one with my position [abortion/gun control].
3. I feel immersed in my position on [abortion/gun control]
4. I have a deep emotional bond with my position on [abortion/gun control].
5. I am strong because of my position on [abortion/gun control].
6. I'll do more for my position on [abortion/gun control] than anyone else.
7. I make my position on [abortion/gun control] strong.

Moral Mandates (scale from 0 (*Not at all*) to 4 (*Extremely*); coded as values between 1-5)

1. To what extent do you feel your position on [abortion/gun control] is based on strong personal principles?
2. To what extent do you feel your position on [abortion/gun control] is a moral stance?
3. To what extent do you feel your position on [abortion/gun control] is morally correct?
4. How much are your feelings about your position on [abortion/gun control] connected to your core moral beliefs and convictions?
5. To what extent are your feelings about your position on [abortion/gun control] deeply connected to your fundamental beliefs about ‘right’ and ‘wrong’?

Sacred Values (scale from 0 (*Completely Disagree*) to 6 (*Completely Agree*); coded as values between 1-7)

1. My position on [abortion/gun control] is something that I should not sacrifice, no matter what the benefits (money or something else).
2. My position on [abortion/gun control] is something which one cannot quantify with money.
3. My position on [abortion/gun control] is non-negotiable.
4. My position on [abortion/gun control] is inflexible no matter what.

Outcome Measure

Sacrifice for Cause (scale from 0 (*Completely Disagree*) to 6 (*Completely Agree*); coded as values between 1-7)

1. I would fight someone threatening my position on [abortion/gun control].
2. I would I would fight someone insulting or making fun of my position on [abortion/gun control].
3. I'd do anything to protect my position on [abortion/gun control].
4. Hurting other people is acceptable if it means protecting my position on [abortion/gun control].
5. I would help others get revenge on someone who insulted my position on [abortion/gun control].
6. I would sacrifice my life if it saved my position on [abortion/gun control].
7. I would sacrifice my life if it advanced my position on [abortion/gun control].

Attention Check Items

Correct responses are highlighted below. Multiple choice responses appeared in randomized order in the survey. Participants who answered the multiple-choice questions incorrectly or failed to rewrite the sentence in Question 1 accurately were excluded from analyses.

1. Please rewrite the following sentence in all capital letters (all caps):

Dan went to the store to buy fruit.

1. Where did Dan likely go?
   1. Grocery Store
   2. Clothing Store
   3. Furniture Store
2. What did Dan buy?
   1. A type of food
   2. A pair of shoes
   3. A piece of furniture

Question 4 appeared on its own page and its response options were not randomized, they were shown in the survey in the order below.

1. When was the last time you cured cancer?
   1. Yesterday
   2. 5 weeks ago
   3. 7 years ago
   4. Never
   5. All the time

Demographic Items

1. What is your gender?
   1. Male
   2. Female
   3. Other (fill-in-the-blank appeared if participants chose this option)
2. What is your age? (fill-in-the-blank)
3. What is your ethnicity (fill-in-the-blank)
4. What country do you consider to best represent your nationality/citizenship? (fill-in-the-blank)
5. What is the highest level of school you have completed?
   1. Less than high school degree
   2. High school degree or equivalent (e.g., GED)
   3. Some college but no degree
   4. Associate degree
   5. Bachelor degree
   6. Graduate degree
6. How do you identify yourself politically?
   1. Liberal
   2. Conservative
   3. Libertarian
   4. Independent
   5. Other (fill-in-the-blank appeared if participants chose this option)
7. How would you describe yourself?
   1. Very Liberal
   2. Liberal
   3. Slightly Liberal
   4. Slightly Conservative
   5. Conservative
   6. Very Conservative
8. What is your religion? (fill-in-the-blank)
9. How religious would you say you are?
   1. Not religious at all
   2. Slightly religious
   3. Moderately religious
   4. Very religious
   5. Extremely religious

**SOM-7 Full Text of Manipulations**

**Study 2 Manipulation** (2 conditions) –

Self-Affirmation Condition (adapted from twenty statements test): First participants were prompted to “Please take a few minutes to fill in the blanks in the following sentences by describing yourself. Write whatever comes to mind. For example, one way that you could complete one of the following ‘I am a’ statements is with the word ‘student’, making it say "I am a student". Feel free to write multiple words.”

Then on the next page participants were told to “Please take a few minutes to describe in more detail why you chose to fill in the previous ‘I am a...’ statements with the words that you chose. As a reminder, in response to ‘I am a’ you wrote…” at which point we piped in their responses from the previous page.

Control Condition: First participants were prompted to “Please take a few minutes to fill in the blanks in the following sentences by describing fish. Write whatever comes to mind. For example, one way that you could complete one of the following ‘Fish are’ statements is with the word ‘wet’, making it say ‘Fish are wet’. Feel free to write multiple words.”

Then on the next page participants were told to “Please take a few minutes to describe in more detail why you chose to fill in the previous ‘Fish are...’ statements with the words that you chose. As a reminder, in response to ‘Fish are’ you wrote…” at which point we piped in their responses from the previous page.

**Study 3 Manipulation** (2 conditions) –

Self-Affirmation Condition: Participants in the self-affirmation condition were prompted to “Please take a few minutes to write about what comes to mind when you think about your death. Please focus on (1) the most personal goals and dreams you'll have hoped to accomplish before death and (2) the legacy that you hope to leave behind. Be as specific or general as you would like.”

Control Condition: Participants in the control condition were asked to write about fish -“Please take a few minutes to write about fish and anything that comes to mind regarding them. Be as specific or general as you would like.”

**Study 5 Manipulation** (2 conditions) –

Personal Self Condition: “Please take 2 minutes to tell us about yourself. Imagine yourself with your closest friend and your friend asks you “What makes you “you”? Imagine your friend isn’t interested in superficial qualities and really wants to know about your enduring, deepest self. ”

Distractor Condition: “Please take 2 minutes to give your opinion about whether there is intelligent life in the universe other than on Earth.”

**Study 6 Manipulation** (3 conditions) –

The study was conducted in two phases to enhance the plausibility of the feedback manipulation. In phase one we measured the three predictors (sacred values, moral convictions, and identity fusion) with respect to the abortion cause. One week later participants received an email inviting them to complete phase 2 of the study, to which they responded within 1 to 39 days. In phase 2, participants received the feedback manipulation followed by the measure of willingness to fight and die for the abortion cause. We introduced the feedback manipulation by leading participants to believe that, based on their responses during phase one, they had been evaluated by a group of psychologists who has prepared an individual report on each of the participants. Participants were told that at the end of the study they would receive the full report, but they could read a brief summary of the main findings at that moment.

Self-discrepant Condition: “In phase 1 you completed several scales. These scales provide two types of information: how you see yourself (which is reflected in the answers you gave) and how you really are (which is inferred from the time it takes to answer each question, if you changed your mind, the incongruity between the answers, etc.). Psychologists have compared these two types of information: 1) how you see yourself and 2) how you really are in five dimensions: shyness, insecurity, stubbornness, nervousness and distrust. In your case, in particular, psychologists have concluded that **the way you see yourself DOES NOT match how you really are in four of the five dimensions evaluated.”**

Verifying Condition: “In phase 1 you completed several scales. These scales provide two types of information: how you see yourself (which is reflected in the answers you gave) and how you really are (which is inferred from the time it takes to answer each question, if you changed your mind, the incongruity between the answers, etc.). Psychologists have compared these two types of information: 1) how you see yourself and 2) how you really are in five dimensions: shyness, insecurity, stubbornness, nervousness and distrust. In your case, in particular, psychologists have concluded that **the way you see yourself matches how you really are in four of the five dimensions evaluated.”**

Control Condition: “The system is taking longer than expected. Please continue with the rest of the questionnaire while we look for your summary.”

After the feedback manipulation, participants completed the outcome measure, willingness to self-sacrifice for the abortion cause.
